# The political economy of expedience: examining perspectives on military support to Sierra Leone’s Ebola response

**DOI:** 10.1186/s13031-023-00553-6

**Published:** 2023-11-06

**Authors:** Samuel T. Boland, Dina Balabanova, Susannah Mayhew

**Affiliations:** 1 Centre for Universal Health, Chatham House, 10 St James’s Square, London, SW1Y 4LE UK; 2 Department of Global Health and Development, London School of Hygiene & Tropical Medicine, 15-17 Tavistock Square, London, WC1H 9SH UK

**Keywords:** Sierra Leone, Ebola, Securitisation, Militarisation, Public health emergencies, Civil-military

## Abstract

The 2013–2016 West Africa Ebola Epidemic is the largest outbreak of Ebola in history. By September, 2014 the outbreak was worsening significantly, and the international president of Médecins Sans Frontières called for military assistance. In Sierra Leone, the British and Sierra Leonean militaries intervened. They quickly established a National Ebola Response Centre and a constituent network of District Ebola Response Centres. Thereafter, these inherently militarised centres are where almost all Ebola response activities were coordinated. In order to examine perspectives on the nature of the militaries’ intervention, 110 semi-structured qualitative interviews were conducted and analysed. Military support to Sierra Leone’s Ebola response was felt by most respondents to be a valuable contribution to the overall effort to contain the outbreak, especially in light of the perceived weakness of the Ministry of Health and Sanitation to effectively do so. However, a smaller number of respondents emphasised that the military deployments facilitated various structural harms, including for how the perceived exclusion of public institutions (as above) and other local actors from Ebola response decision making was felt to prevent capacity building, and in turn, to limit resilience to future crises. The concurrent provision of life-saving assistance and rendering of structural harm resulting from the militaries’ intervention is ultimately found to be part of a vicious cycle, which this article conceptualises as the ‘political economy of expedience’, a paradox that should be considered inherent in any militarised intervention during humanitarian and public health crises.

## Introduction and background

The 2013–2016 West Africa Ebola Epidemic was and remains the largest outbreak of Ebola in recorded history. For several months following this initial case, the outbreak was mostly limited to rural areas. However, in the summer of 2014, the crisis escalated, and the World Health Organisation (WHO) declared a Public Health Emergency of International Concern (PHEIC) [1].

On September 2nd, 2014, the then-International President of Médecins Sans Frontières (MSF) released a statement that was uncharacteristic for the international non-governmental organisation ((I)NGO): that it would take military mobilisation to bring the situation under control [2]. In Sierra Leone, the British Armed Forces deployed shortly thereafter alongside the Republic of Sierra Leone Armed Forces (RSLAF). To some extent (and as reflected on later in this article), this was in keeping with recent history, wherein the United Kingdom (UK) helped reform RSLAF in the years following the 1991–2002 Sierra Leone Civil War as part of the International Security Sector Advisory Team (ISAT) programme. This reinforced close post-colonial relationships between Sierra Leone and the UK, and also ensured the national military was relatively well funded compared with other public institutions.

Military support included the establishment of the National and District Ebola Response Centres (the NERC and DERCs, respectively) in late 2014, and the performance of various command and control (C2) functions within these centres [3]. This replaced a wholly civilian response organ, the Ebola Operations Centre (EOC), that had been jointly led by the Ministry of Health and Sanitation (MoHS) and the WHO [3]. Military contributions to Sierra Leone’s Ebola response were therefore a core component of the overall response.

In light of the unfolding crisis, many perceived the militaries’ intervention to be a necessary ‘last resort’ (this was evident, for example, in the aforementioned statement by MSF’s International President) [3–6]. To some scholars and humanitarian practitioners, however, such a substantial and conspicuous military intervention during peacetime in response to a public health emergency is cause for concern (especially in a country that had recently experienced civil war) [7–14]. Relatedly, others have criticised the use of militaries in this way as a case of the inappropriate ‘securitisation’ or ‘militarisation’ of civilian domains that should be guided by the Humanitarian Principles, as well as for the ways it risks usurping civilian leadership; causing unnecessary alarm or confusion amongst plausibly traumatised populations; and perpetrating—or at least threatening the possibility of—human rights abuses and other forms of violence at the hands of armed actors that are foisted onto vulnerable communities in the name of public health [7, 8, 15–18]. Further, other scholars have argued that civilian response and military actors are organised in such different ways that there are inherent and perhaps insurmountable challenges to their ability to effectively and productively cooperate in response to humanitarian crises and public health emergencies [19–21].

Due to the importance of the militaries’ intervention in Sierra Leone and also the broader debates around its appropriateness, this article seeks to document and examine perspectives on the nature of the militaries’ intervention that followed as perceived by Ebola response workers (ERWs).

## Methods

### Ethics

Ethical approval was granted by the London School of Hygiene & Tropical Medicine (LSHTM) Research Ethics Committee (reference #14,424) and the Sierra Leone Ministry of Health and Sanitation (MoHS) Office of the Sierra Leone Ethics and Scientific Review Committee (no reference number; approved 28 August 2017). All research was conducted according to accepted norms for ethical research, including the documentation of informed consent; the confidentiality of participation; and the anonymisation of statements provided.

### Data collection

**Fig. 1:**
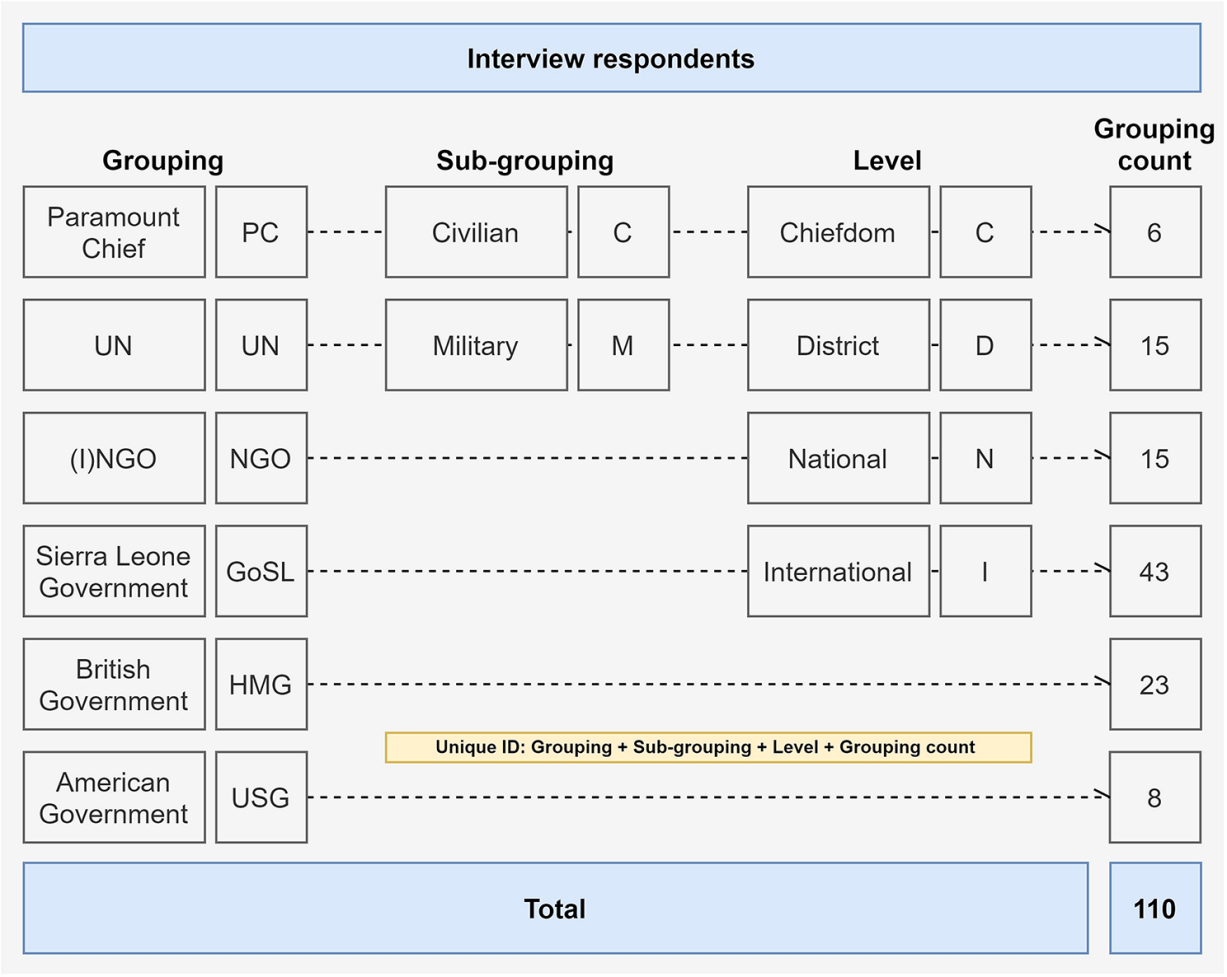
Interview respondents. (Source: author)

110 semi-structured qualitative interviews were conducted over two years (2017–2018). A wide array of civilian and military ERWs at Sierra Leone’s chiefdom, district, and national levels were targeted, as were those at the international level (Fig. 1). All interviews were conducted in English by lead author (SB).

Three specific research sites were chosen: Kambia District (for chiefdom and district-level perspectives including at the DERC); Port Loko District (to complement Kambia District data where saturation had not been met); and Western Area Urban District (for national-level perspectives). Sites were selected due to SB’s prior work in these areas, facilitating a greater level of access than might otherwise have been possible (though this also introduces possible biases, reflected on in the limitations section of this manuscript). A general snowballing technique was used. Data collection continued until saturation had been met. Each respondent was designated a unique identifier (ID) according to their grouping; sub-grouping; level; and count (assigned sequentially), as described in Fig. 1. Interviews were audio recorded and transcribed. They were conducted using a topic guide; despite the topic guide, interviews were conversational and open-ended in nature, so as to include new themes and avenues for exploration as relevant including consideration of relevant social and political histories.

### Analysis

Interview data were organised using framework analysis. Familiarisation was accomplished through the Principal Investigator (PI) role conducting all interviews (limitations of this are reflected on later in this article). Once data collection was complete, a code frame was developed in NVivo to process transcripts. Sheikh et al.’s ‘systems software’ and ‘systems hardware’ distinction (2011) was used to help map references to the various contributions made by the militaries following their respective deployments [22].

This was selected inductively, as initial grounded theory coding was later found to conform to this typology, which was itself useful for differentiating concrete ‘hardware’ contributions made by the militaries compared with more intangible ‘software’ contributions. However, it was also found to be a powerful analytic vehicle for elucidating some of the more complex findings in the data. Systems software is defined by Sheikh et al. as “ideas and interests, values and norms, and affinities and power that guide actions and underpin the relationships among system actors and elements” [22]. Therein, the framework helped to facilitate analytic method beyond a positivist one, to consider political and social contexts (actively explored with respondents as described above) and therein to explore how respondents’ values, motives, and entanglements might affect not only their perspectives but also the overall effect of the militaries’ intervention in Sierra Leone more generally speaking.

All qualitative interviews were coded using this methodology. Then the code frame was examined and nodes removed or (dis)aggregated where appropriate. Relationships between recurring themes were then charted, mapped, and interpreted for patterns and variations between research groupings. Findings were shared with all respondents.

### Limitations

Site selection was limited to the Western Area Urban, Port Loko, and Kambia districts of Sierra Leone. These areas were affected later in the Ebola outbreak, after lessons had been learned and more resources made available. Respondents’ perception of the Ebola response—including the militaries’ contributions—may therefore be more positive than elsewhere. The areas are also Temne- and APC-majority areas (being the government in power at the time), suggesting perhaps a relatively privileged perspective which may affect governance issues. These locations were chosen due to SB’s prior work there during the Ebola response, with initial subject selection relying on those known to SB (themselves a civilian Ebola responder originally from the United States). This resulted in a number of plausible strengths to the research, inasmuch as there was core knowledge and existing trust, but also introduces the possibility of various interviewer and other biases. It is plausible, for example, that a Sierra Leonean interviewer may have elicited different responses than those that were documented. As with other biases, the effect of this is limited by the large and diverse number of respondents, the vast majority of whom were identified through snowballing (i.e., not previously known to SB). There is an under-representation of female respondents (21.8%) across respondent groupings, reflecting the general skew of those who worked in the NERC and DERCs, but may nevertheless limit generalisability. Local actors made significant ad-hoc contributions to Sierra Leone’s Ebola response (i.e., informally outside the NERC and DERCs), but the perspectives of these actors were not systematically documented for this study. Relatedly, in much smaller deployments, other militaries (e.g., the Chinese and Irish) were deployed to Sierra Leone outside the NERC and DERCs, [23, 24] which was not captured in the data. Also uncaptured are the experiences in the other effected countries, namely Liberia and Guinea, which were beyond the capacity of the research team and therefore the scope of the research project—examining these questions in those countries would represent an invaluable additional perspective to complement and better nuance the findings identified in Sierra Leone.

Limitations were mitigated through the large number of respondents and their diversity; the confidentiality of participation and anonymisation of statements provided; and reflexive best practice.

## Results

Drawing primarily on national and district respondent perspectives, the perceived nature and effect of the militaries’ intervention is examined, considering first the benefits that many respondents felt it may have effected, and then some of the harms it may have facilitated.

### Military commendation

Following their initial intervention in the late summer and autumn of 2014, respondents noted various systems hardware and systems software contributions by the British Armed Forces and RSLAF to Sierra Leone’s Ebola response.

### Military systems hardware

**Fig. 2:**
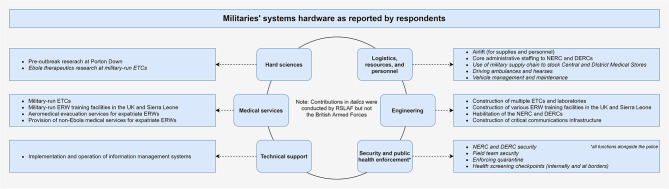
Content of role and support provided by militaries to the Sierra Leone Ebola response as reported by respondents. (Source: author)

Derived inductively from respondents’ contributions, systems hardware support included: contributions to the hard sciences; medical services (particularly by RSLAF at the Police Training Centre and 34 Military Hospital); technical support; logistics, resources, and personnel; security and the enforcement of public health measures including quarantine; and engineering (Fig. 2). Notably, with one limited exception discussed later in this article, the UK’s deployment did not include the kind of large-scale deployment of military medics to ETCs that was anticipated by MSF when they made their infamous request in September 2014. This was due to force protection requirements decided at the political level that limited the UK’s willingness to put soldiers in harm’s way [25–27]. Further detailing each of these systems hardware contributions is beyond the scope of this article (they are also described elsewhere), [4] but they were nevertheless referenced frequently by respondents and collectively represent an important component of the militaries’ intervention.

### Military systems software

Respondents also noted several positive aspects of the militaries’ intervention which were related to a way of working and organisational culture, including an agreeable military manner, mindset, and *modus operandi*—i.e., the militaries’ systems software (also derived inductively, Table 1).

**Table 1:** Militaries’ systems software as reported by respondents

Description of the military form that was perceived by respondents to represent a positive contribution	Frequency (/110)
Compatibility with civilian ERW working culture; positive and complementary civil-military relationships (CMRel)	96
General sense of professionalism; technical expertise	56
Making others feel safe and secure (as distinct from providing security per se)	43
An apolitical nature (note: while 11 represents a small proportion of the overall number of respondents, it is significant, due to the fact that 55 respondents specifically criticised various civilian ERWs for prioritising politics or business interests over containing the outbreak, or, for being engaged in some kind of corrupt practice. This is despite the fact that respondents were not explicitly asked to speak to civilian ERW limitations. Therein, this finding contrasts significantly with how respondents often characterised civilian ERWs)	11
Exhibiting friendliness, humanity, and empathy	49
Flexibility	19
Skills in C2; organisation; planning; preparedness	85
Acculturating discipline; efficiency, time management, and focus; a hard-working attitude; and ‘just getting on with it’	71

In other words, most respondents noted that the militaries delivered not just goods and services, but also embodied a certain way of working that many found compelling given the urgent circumstances.

### Creation of an enabling environment

The militaries’ systems hardware and systems software contributions were perceived by respondents to have various important and positive effects on the Ebola response. Of significant note—particularly amongst respondents who were present in the outbreak before the transition to the military-led NERC and DERCs—is the way the militaries were felt to create an enabling environment in which civilian ERWs could more safely, significantly, and effectively intervene.

This was particularly the case for the Ebola and non-Ebola medical backstop the militaries provided to national and expatriate ERWs (with some services reserved for the latter): as described by respondents, the British Armed Forces built and staffed the Kerry Town Treatment Unit (KTTU) to provide Ebola medical care to national and expatriate ERWs (HMG-M-N-6); and made the Royal Air Force’s (RAF’s) Deployable Air Isolator Team (DAIT) available for the evacuation of the latter to Britain for tertiary care (HMG-C-N-13). Taken together, respondents generally agreed that these services ensured the provision of both Ebola and non-Ebola medical care to ERWs (for expatriate ERWs in particular), which in Sierra Leone, was not otherwise dependable or of high quality. Without these guarantees facilitated by the British Armed Forces, recalled an HMG civilian respondent, “*the reality was it was a real struggle to get NGOs to come and operate*” in response to the Ebola outbreak in Sierra Leone (HMG-C-N-14).

The most significant effect of these specific interventions was not, per se, for the delivery of healthcare services directly (for example, only a small number of ERWs were eventually treated in KTTU (USG-C-I-5), and almost none received care at RFA Argus (USG-C-N-3)). Rather (and as to be examined at length), it was for the assurance—both psychologically and logistically—that the availability of these services was perceived to effect. Many civilian ERW respondents who were either present in Sierra Leone in the autumn of 2014 or considering deploying to the response around this time echoed this notion (especially expatriate respondents, because as above, many of these military-supported healthcare services were reserved for them; further, they had considerably more choice over whether to intervene in the Ebola response when compared with national healthcare workers). For example, an (I)NGO respondent recalled how

*…even if the British didn’t do anything, the very fact that they were there… had a psychological effect on international agencies who suddenly felt a lot more confident and safe… For me, personally, it probably was quite significant… Suddenly… it felt like the cavalry had arrived, and if things got really bad, there would be someone there who had my back… That probably made me more confident and more willing to take on more things… So, I think there’s… a psychological element… And the KTTU [UK] MoD unit… [was] part of that (NGO-C-N-10).*

In other words, even if the services were never used, the simple presence of the British Armed Forces was felt by this respondent to contribute to a sense of security, so that if and when the situation deteriorated (as it plausibly could), an institution was present that was empowered to support them as needed.

This was echoed by another (senior) USG civilian respondent who was responsible for approving the deployment of their agency’s staff to Sierra Leone:

*The role of militaries… at [KTTU]… was a good idea… I was talking to my friends and… to my staff, and saying, I’d like you to do this [i.e., to deploy to Sierra Leone]. Would you be willing? Let’s have a serious conversation right now. There’s Ebola transmission happening in the community, and you have to take that into account when you’re making your decision. You might be exposed, even if you do everything cautiously and carefully… There was a real risk to our deployers… Being able to say that there’s a level of medical care that is higher than what you generally expect… to be run [in-country]… for people that [are]… proactively putting themselves in the line of fire? That was a really important element… that was very useful and important to the success of the response because… [of] that reassurance (USG-C-I-5).*

To this respondent helping decide whether or not to deploy their staff to Sierra Leone, therefore, this sense of assurance was very significant, as it was to many other civilian ERW respondents. Importantly, it was also an intentional effect of the British Armed Forces’ intervention, made to encourage (primarily international) NGOs—many of which (as above) had either evacuated their staff from Sierra Leone, or had not yet intervened in response to the crisis—to consider deploying.

“*That is what the plan was*”, said a British Armed Forces respondent involved in initial high-level planning meetings, who continued:

*The backstopping [of the] international community, to say, ‘you can all come and help, all you humanitarians, come and do your job, we will make sure [that] if the worst comes to worst, we will look after it’. Not just Brits, but Italians, Spanish, and Germans and Dutch and whatever… That was the military goal… [and] contribution, to safeguard the UK interest and ensure that all [the] NGOs will continue working, because we would backstop [their] primary healthcare and [their] Ebola healthcare… That’s why DfID said ‘it’s worth it, otherwise we are not going to get these other people’ [to come] (HMG-M-N-5).*

Various other high-level respondents involved in the decision to deploy RFA Argus, establish KTTU, and commit the DAIT agreed that the primary effect these contributions were intended to have was not the provision of care itself, but a guarantee to civilian ERWs that it was available to them were it to be required (HMG-M-N-6; HMG-C-N-13; HMG-C-N-14; USG-C-N-3; HMG-M-I-1; HMG-M-D-4; HMG-C-I-9; USG-M-I-1).

For example, as recalled by a senior HMG civilian respondent with responsibility for helping to secure HMG funds for these military interventions, “*you couldn’t have confidence that any other international actor including NGOs would stick with it, without a guarantee… [that] a government as a sovereign state actor would ultimately underwrite [the risks]*” (HMG-C-I-9).

It was, put simply by a British Armed Forces respondent, a military

*…mission… that [they] made explicit… to boost international confidence so that more people would come and fight Ebola, and… be prepared to get into it and get their hands dirty with it, with confidence that we would look after them (HMG-M-N-6).*

In addition to these assurances, both militaries provided important training to ERWs. For example, in the autumn of 2014, medics from both RSLAF and the British Armed Forces established an Ebola Training Academy at the University of Sierra Leone’s College of Medical and Health Sciences (COMAHS). Over the course of several weeks at this (and one other) centre, military personnel trained 4,200 Sierra Leoneans in biohazard protections to fill roles across the burgeoning Ebola response (GoSL-C-D-1; GoSL-C-N-20; NGO-C-N-6; NGO-C-N-10). Meanwhile, in the UK, the British Armed Forces built mock-up Ebola Treatment Centres (ETCs) within their Medical Services Training Centre, where they then trained the various civilian emergency medical teams preparing to deploy to Sierra Leone (NGO-C-N-6).

These trainings were primarily focused on the appropriate use of personal protective equipment (PPE) and infection prevention, which did draw some criticism from a small number of respondents for the ways that guidance to ETC healthcare workers (HCWs) was focused on avoiding contact with patients. For example, one (I)NGO respondent recalled how the militaries

*…were training something called no-touch care, in which they were basically telling health professionals not to touch patients. And this caused a huge amount of upset amongst Sierra Leonean health professionals, who were saying ‘These are our community members, these are our families, what do you mean don’t to touch them? We want to give them IVs [i.e., intravenous therapy], we want to… care for them’… [The military trainers] didn’t do any training in providing clinical care, it was all about how to take on and take off your gear and not get exposed (NGO-C-N-10).*

However, for the thousands of other ERWs that were to perform non-healthcare roles that nevertheless required PPE—such as ETC hygienists, decontamination workers, ambulance drivers, and burial team workers—this military-supported training was considered by many to be crucial (civilian ERW respondents noted other forms of training they received from military personnel, such as one who received training in both information technology and vehicle fleet management (GoSL-C-N-20), and another who received training in geographic information systems (USG-C-N-3)). These newly trained ERWs were made available to staff the ETCs being built throughout the country by the British Armed Forces’ Corps of Royal Engineers, which were opened to Ebola patients towards the end of 2014 and the beginning of 2015 (HMG-M-N-5).

Taken together, over the first few months of their intervention, the militaries put in place Ebola and non-Ebola healthcare infrastructure for ERWs (facilitating their intervention); built a number of ETCs for the care of Sierra Leonean civilians; and established and ran training programmes so the latter could be more robustly and safely staffed. As summed up by an HMG civilian respondent, this was “*quite catalytic to [the] broader response… [which therefore] had a disproportionate impact in terms of enabling the rest of the response*” (HMG-C-N-13).

As described by respondents, an enabling environment was thus created, in which ERWs could more concertedly and safely intervene.

### Effecting a clarity of purpose

Thereafter, one of the most cited positive effects of the militaries’ intervention coalesced around the notion that they worked in a way which was different—in some ways preferably so—to many civilian ERWs. Some respondents had difficulty putting this intangible effect into words, such as an (I)NGO respondent who stated:

*I can’t really say like, ‘oh, here are the pieces of [why the militaries’ intervention was so important]’, but essentially, it’s just really small things that made the operations more efficient. Just like, a different mindset, you know? Just like… stronger, pragmatic operations that are based on efficiency and getting shit to work (NGO-C-D-12).*

In other words, this respondent felt that the military “*mindset*” represented a different way of working to civilian ERWs, that they thought strengthened day-to-day Ebola response activities through better aligning and operationalising them (NGO-C-D-12). Many respondents recalled this positive shift as resulting from a clarity of purpose that was effected through the application of the militaries’ mindset, which was not felt to have characterised the Ebola response prior to their intervention. For example, one (I)NGO respondent (notably, one that was atypically critical of the militaries’ intervention in much of their interview) recalled how the EOC-led response was characterised by “*political meddling… [and] corruption… [The militaries] came in and created a kind of rules-based system… Up until that point, it was complete smoke and mirrors*” (NGO-C-N-10).

One UN respondent described it simply as “*refreshing [to have] predictability and reliability and accountability*” that they felt had not been present before (UN-C-N-3). As above (Table 1), while a small number of respondents explicitly cited an apolitical nature as a positive attribute of the intervening militaries (n = 11), a large number (across all respondent groupings) specifically criticised the way that some civilian ERWs prioritised political, business, and other personal interests over the objective of containing the outbreak (n = 55). This was noted despite respondents not being asked to speak to the limitations of civilian actors during interviews.

One HMG civilian respondent argued that this difference between civilian ERWs and the involved militaries was because, for the militaries, “*there was no… political imperative other than ‘how do you help stop this potentially ravaging outbreak as quickly as possible?’… It was simple as that*” (HMG-C-N-14). They suggest, in other words, that the militaries’ focus was on their given (and civilian-defined) mission objective, which this respondent felt was relatively unambiguous: end the outbreak.

While the Sierra Leonean military has a more distant history of involvement in national politics, respondents noted a relative lack of corruption and “*political meddling*” (NGO-C-D-10) compared to what they had seen under MoHS leadership—though respondents associated this not, per se, because the military institutions or their personnel were thought to be more inherently ethical, but rather is because of incidental factors.

For example, many military respondents attributed this perceived difference to the fact that their salaries did not depend on the length of the outbreak (i.e., that the militaries’ incentive structure for personnel was not designed to favour the prolonging of the crisis); further—unlike a number of UN agencies and (I)NGOs—there was also no obvious institutional benefit to the militaries if the outbreak persisted (USG-M-I-1; HMG-M-D-8; GoSL-M-D-5). Relatedly, several other respondents noted the high financial cost of the militaries’ intervention, and how this meant there was a strong interest within HMG to wind down Operations Gritrock and Octopus as expeditiously as possible (HMG was also financing RSLAF’s Ebola response contributions) (HMG-M-N-7; HMG-M-N-9; HMG-C-D-6; HMG-C-D-7; HMG-C-D-2; HMG-C-N-13). On this basis, in the words of one senior military respondent, as soon as a military is deployed, “*they automatically start planning their exodus*” (USG-M-I-1), which in this case meant accomplishing their mission objective of containing the outbreak as efficiently as possible (several respondents nuanced the idea that the militaries’ intervention was very expensive, suggesting instead that when one considers the inestimable financial benefit of the enabling environment that was created, the high costs associated with the militaries’ intervention may have actually been economical).

### Civil-military complementarity

Findings show that the militaries were felt by many respondents to help effect a clarity of purpose, which was applied structurally within the new NERC and DERCs: regular meetings were instituted, as were SOPs and delineated scopes of work; further, lines of accountability were made explicit, previously fractured information systems were harmonised, and focal points were identified for the growing number of workstreams constituting the response’s bespoke ‘pillar system’. Through the NERC and DERCs, in other words, the militaries were felt to “*put all these elements together*” (HMG-C-D-6) under necessary accountability mechanisms. Perceived efficiency rose considerably.

Through their interventions within and leadership over these structures, the militaries were felt by a significant majority of respondents (n = 96) to complement, rather than usurp, the interventions of their civilian counterparts, especially through the application of their perceived strengths in C2, organisation, preparedness, and planning (n = 85) (though there are several important dissenting views, which are addressed later in this article). For example, a senior UN respondent recalled being in a high-level meeting with military personnel where the strategic plan to contain the Ebola outbreak in Sierra Leone was being developed:

*And then it hit me… What everybody forgets about military is that they bring in planners… So, [I was talking with] these [military] guys [in the room and outlining a strategic plan]… their faces lit up, and they were laughing, and I said, ‘so guys, like, what stupid thing did I just say?’ They said, ‘well, you’re the first [civilian] that sounds like us, and we understand what you’re saying.’ It was very funny, [and] then we started talking about what… the response needed to look like (UN-C-I-13).*

This convivial civil-military exchange evidences how—unlike the intervening militaries as above—civilian ERWs were not thought by some to have particular strengths in planning, which was corroborated by a majority of respondents (n = 59) across all groupings.

Accordingly, military ERWs often prided themselves on their relative capacity for the “*operationalisation of… [civilian ERWs’] nebulous ideas into day-to-day actions*” (USG-M-I-1); and civilian ERWs often appreciated them for it, because they could effectively “*turn a [civilian ERW’s] policy into an implementation plan*” (NGO-C-N-7). Regardless of research grouping, most agreed, such as one British Armed Forces respondent who recalled how the DERC’s military personnel

*…would lead… [but] try to do so in a way where everybody in the [DERC evening brief’s] audience knew that they were playing a part, and that they [were] a part of the decision making process… And… [then they would work to] just bring… together every brilliant idea and put it into a plan (HMG-M-D-4).*

In other words, the DERCs’ military personnel were generally perceived to be able to bring different ideas and areas of expertise together and align them in a collaborative way (another British Armed Forces respondent echoed this catalysing notion in a more cynical way: they stated that “*where the military really added benefit was to stop people navel gazing about how to respond and just to get on and respond*” (HMG-M-N-2), suggesting civilians focused too much on discussing how to respond rather than actually responding). One military respondent used Plato’s Allegory of the Cave to nuance this idea, describing how they felt that military ERWs were not only effective planners, but were also able to help civilian ERWs to move past their “*very focused way of looking at the world… [by] forc[ing] them to step out of the cave and not just look at the shadows… [which] help[ed] build that… common operating picture*” (USG-M-I-1).

In other words, this respondent felt that the intervening military personnel were able to complement civilian ERWs’ technical and medical expertise (being applied within specific pillars) by ensuring interventions considered other important operational and logistical factors. This was variously described by civilian and military respondents alike as military “*muscle*” (GoSL-C-N-17) and “*logistics*” (GoSL-C-N-26) as a counterpart to civilian “*brain*” (GoSL-C-N-17; NGO-C-N-6), “*expertise*” (NGO-C-N-7), and “*technical knowhow*” (GoSL-C-N-26). The way in which this civil-military complementarity was perceived to facilitate civilian ERW interventions was effectively captured by a GoSL civilian respondent (notably, a high-level stakeholder and member of the response’s leadership), who stated:

*I find it quite hypocritical and frankly endlessly condescending to say to me that in my country, when we had 163 doctors for a population of 6 million people… that we shouldn’t bring in the military to help us organise and plan… Let us not forget that the army… just helped us [civilians] to organise and plan… with a kind of coherence… Before [the military arrived], doctors were up at 3:00am trying to find fuel to put in an ambulance… [We] didn’t want [our] doctors to be worrying about where fuel was going to come from for the ambulance to take their patients. I just want[ed] the doctors to say, ‘This patient should be taken to [the ETC]’ (GoSL-C-N-17).*

The militaries, therefore, were felt to take on and address a number of the logistical problems that were consuming the time and attention of medical experts who could otherwise have focused on case management and patient care (this argument also applies to any other health or public health expert who was limited by these same constraints). A British Armed Forces respondent also noted how “*civilian organisations [also] recognised the ability of the… military to organise and create that structure within which they could do their job*” (HMG-M-D-4).

A further key point is how a majority of military respondents recognised their own weaknesses in responding to the Ebola outbreak (i.e., the ways this civil-military complementarity was reciprocal in nature). For example, the same British Armed Forces respondent acknowledged that civilian ERWs “*have medical expertise and the humanitarian knowledge*” that military personnel do not, but that the militaries’ superior organisational skills could be “*combine[d and applied together] for the greater good*” (HMG-M-D-4). The analysis thus suggests the plausibility of closer alignment between military and medical approaches than might be typically assumed.

### Military-military complementarity

A smaller number of respondents—primarily military respondents, because they were more directly affected and also more aware of the relevant history—also referenced how valuable they found not only civil-military complementarity, but also military-military complementarity resulting from relationships which were built between the British Armed Forces and RSLAF through the post-civil war ISAT programme. Respondents usually characterised this complementarity as relatively one-sided in nature (i.e., that it was felt the presence of the British Armed Forces was valuable for making RSLAF a better civil-military partner).

For example, several non-Sierra Leonean respondents felt that the presence of the British Armed Forces alongside RSLAF meant that concerns related to the latter’s role in the Ebola response could be more efficiently and effectively addressed (HMG-M-N-5; HMG-C-I-9; HMG-C-D-6; UN-C-I-13), with one HMG civilian respondent recalling how there was the

*…ability for an officer from the British military to basically go and close the door and say to a senior commander in the RSLAF ‘this is wrong’… You [were] sort of leveraging this kind of traditional respect for the British military [in the country]… That is the stuff that would be hard to contract out (HMG-C-I-9).*

In other words, the respondent felt that the close history between the two militaries meant the British Armed Forces was able to hold RSLAF to account, in a way that could not have easily been delegated to a civilian agency or other partner.

On this basis, one senior UN respondent said the British Armed Forces was

*…the bridge that [was]… critical to make this response work… The degree of [military-military] integration was completely different [when compared with the other Ebola-affected countries]… There was a professional military-to-military respect… and none of the arrogance… that I sense elsewhere (UN-C-I-13).*

The depth of the British Armed Forces’ relationship with RSLAF meant HMG civilians also felt they could “*hold [RSLAF] to account*” as an extension of this military-military relationship (HMG-C-D-6) (though this presumably also resulted from the fact that, as referenced above, HMG bankrolled RSLAF’s Ebola response contributions).

Notably, the RSLAF staff assigned to DERCs were not high-ranking generals, but rather lower-ranking majors and captains. Many, therefore, would not have participated in the 1991–2002 Sierra Leone Civil War, as they would have been too young. Transitively, many had entered a newly professionalised military and had gone through training by the British Armed Forces as part of the ISAT programme (a history of which is reflected on more in the discussion) where they were “*trained by the British with a very strong hand*” (HMG-M-N-5). Therefore, many RSLAF personnel had close working relationships with the British Armed Forces (and, in the opinion of an ISAT-affiliated British Armed Forces respondent, also a professionalism that was *“a pure product of post-civil war British trained officers*” (HMG-M-N-5)).

This is somewhat unique to Sierra Leone, and also incidental to the Ebola response: continued the respondent, “*you can’t [realise that professionalism] in a month, a week, a year… You know, that’s a generation of officers*” in the DERCs that had close training linkages to the UK not replicated elsewhere by HMG (HMG-M-N-5). Because of this, in the words of an HMG civilian respondent, the Sierra Leonean military were “*better organised, better trained, and more respected [than] some of the shambles that go on [elsewhere]*” (HMG-C-D-1). In short, the military-military collaboration found in the ISAT programme, as well as its incidental timing (i.e., that it was put in place and operated over the roughly fifteen years preceding the Ebola outbreak), was perceived by many to be fundamental to the overall success of RSLAF in their contributions to the civil-military Ebola response.

The thoroughly civil-military nature of the response in Sierra Leone means that specifically evaluating the epidemiological impact of the militaries’ contributions is not possible. Nevertheless, through the civil-military coalescence that occurred and the many military contributions that were made—including through the delivery of both systems hardware and systems software assets, which helped to create an enabling environment; effect a clarity of purpose; and realise a complementarity with both civilian and other military ERWs—the outbreak did subside. By the autumn of 2015—one year after the deployment of Operation Gritrock and the creation of the civil-military Ebola response architecture—the epidemic was largely over in Sierra Leone, and Operation Gritrock was stood down. On January 1st, 2016, the NERC and the DERCs also closed, with leadership of remaining Ebola response functions transitioned back to the MoHS. Just two further Ebola cases were identified in Sierra Leone, and the country was declared Ebola-free on March 17th, 2016.

### Military maleffect

While a majority of respondents (across different respondent groups) expressed an overall positive association with the militaries’ intervention in Sierra Leone’s Ebola response, a small but significant minority (n = 13) felt otherwise. Relatedly, a majority of others expressed an overall positive association, but referenced particular negative elements.

Criticism ranged from the use of militarised terminology (n = 19), to a cumbersome and inflexible military bureaucracy (n = 15), to feelings that the militaries should have intervened sooner (n = 18). The five most frequently discussed criticisms, though, related to: instances of coercion; military force protection requirements hindering response work; a lack of relevant expertise; the duplication of work being done (or that could have been done) by civilian ERWs; and CMRel problems either defined generically, or as resulting from incompatible ways of working with civilian ERWs, or being specific instances of harshness or dismissiveness with civilian ERWs (Table 2).

**Table 2:** Most frequently cited drawbacks and other negative associations with the intervening militaries as reported by respondents

Description of the military form that was perceived by respondents to represent a negative contribution	Frequency (/110)
Coercive approaches or attitudes	33
Unnecessary work, waste, or expense	57
Generic problems with CMRel (n = 43); different or incompatible ways of working with civilian ERWs (n = 32); specific instances of harshness or dismissiveness with civilian ERWs (n = 43)	70
Lack of a technical public health perspective or relevant expertise	45
Force protection requirements hindering response work	34

Three key themes were identified: a dismissive military attitude towards civilian ERWs; their interventions’ limited inclusion of local actors; and the obstruction of capacity building amongst public institutions and local actors that could have been enabled were the response organised differently.

Dismissive military attitude.

As above (Table 2), during the course of the Ebola outbreak, a number of civilian ERW respondents (n = 43) noted instances when their military colleagues were dismissive or unnecessarily harsh with either them or other civilian ERWs. A similar number (n = 32) described how a military approach was different to a civilian approach in a way that caused some difficulty. Taken together with other problems (defined generically) with CMRel (n = 43), a majority of respondents (n = 70) felt there were times when CMRel were imperfect (This is not summative, as there is a significant overlap between respondents; further and as above, while a majority expressed instances of imperfect CMRel, a more significant majority nevertheless believed the militaries’ intervention was overall positive in nature and effect).

One GoSL civilian respondent working for the MoHS, for example, suggested that the militaries’ intervention was akin to someone telling you how to clean your own house (i.e., that to be told how to effectively manage one’s domain was deeply patronising) (GoSL-C-N-26). Further, the respondent also described how the military C2 approach that was like forcing a child to eat boiling rice that destroys their nostrils and creates sores (GoSL-C-N-26)—it was felt to be not only demeaning, therefore, but also actively harmful.

At times, respondents noted (sometimes in a positive way) how this amounted to an undemocratic approach. For example, one GoSL civilian respondent stated that

*When you are dealing with civilians…they would always have to talk about democracy. ‘Oh, let us do this’, ‘no, [let us do that]’, you know?… During the time of emergency, you have to give up your own personal individual right as a matter of expedience. So, you cannot come and argue, because it is an emergency (GoSL-C-D-16).*

Notably, therein, the militaries’ approach was often felt to eschew a consensus-based approach (in this case, a perceived-to-be positive effect for the sake of efficiency in response to the emergency at hand). An RSLAF respondent echoed this, stating: “*People would say ‘democracy!’. Yes, democracy, but, if you don’t enforce certain rules, it will not work properly, you see?*” (GoSL-M-N-16).

The way in which dissent was suppressed by the militaries’ approach extended to their enforcement of public health measures including militarised quarantines, wherein military personnel were placed outside of households to ensure that quarantined contacts remained in situ throughout the virus’ 21-day incubation period. While a large number of respondents felt this was inappropriate or potentially harmful (n = 35)—indeed, a small number (n = 11) recalled instances were militaries threatened to use force against or allegedly abused civilian populations—a similar but larger number (n = 41) felt this was a necessary and helpful intervention.

### The insufficient inclusion of local actors

When the militaries intervened in the autumn of 2014, they helped to establish the NERC and DERCs. This had many effects, a number of which were perceived to be positive (as previously described). However, it was also perceived to have some negative effects, including for the way that the new civil-military architecture did not sufficiently consider or systematically include important local actors.

In other words, the militaries’ dismissive attitude that civilian ERWs sometimes perceived (as above) also extended to a kind of structural dismissiveness inherent in the civil-military command centres that were established, in that many individuals and groups with potentially valuable contributions to make were not adequately incorporated. One GoSL civilian respondent summarised this effectively, stating that

*…before the coming of the DERC and even before the involvement of a lot of partners, Sierra Leoneans opted to do [Ebola interventions] on their own… At the village level, town level, [and] section level, we [had] volunteers that were doing it free of charge to save lives and to save people. But when… the command centre came and they started… institutionalising these things… [and] recruiting these people,… the problem comes out… [The DERCs] employed other new people… For those people that were in the villages [and] the towns that were doing these things for nothing—those people know their people, [and] they were left out… And the people never accepted them, and they said, ‘we were doing it for nothing, and now that employment came, you have forgotten [all that we have done]’ (GoSL-C-D-13).*

While these informal responses to the Ebola outbreak were therefore important interventions that mitigated the Ebola outbreak in Sierra Leone, this respondent felt they were never fully understood, incorporated, or capacitated within the militarised NERC and DERCs that were later established (a phenomenon which other scholars have noted) [28–30]. In short, the militaries’ intervention was perceived by some respondents to have “*side-lined and forgot about the experts who were [already] there*” (GoSL-C-N-26).

Notably, all Paramount Chiefs that were interviewed (n = 6) described feeling insufficiently supported in some way during the Ebola response, despite being compelled by the national government to intervene. Half (n = 3) specifically stated that this undermined their local authority, especially for the way that the government expected them to police Ebola bylaws (PC-C-C-1; PC-C-C-2; PC-C-C-6). This included the requirement that Paramount Chiefs issue significant financial penalties to (usually vulnerable and poor) chiefdom constituents that were thought to have contravened the bylaws, which aggravated them (other scholars have noted how the Ebola response misunderstood and homogenised conceptions of local legitimacy; [31] reconfigured local forms of power; [30] and perhaps changed the very nature of local citizenship in Sierra Leone) [32] Paramount Chief respondents also noted the relative lack of funding they were provided to run Ebola response task forces and to conduct other Ebola response interventions (PC-C-C-1; PC-C-C-2), despite the significant sums afforded elsewhere (HMG-M-N-5). Accordingly, one Paramount Chief summed up their time responding to the Ebola outbreak as a sub-district local authority (i.e., below the level that might be fully incorporated within the DERCs) by stating that “*the experience of [being a] Paramount Chief in this Ebola [response] was very pathetic*” (PC-C-C-1).

### Civilian disempowerment

A number of respondents felt the militaries and their (style of) intervention could be dismissive in both attitude and effect to civilian ERWs and other local actors seeking to participate in the Ebola response. Importantly and as above, this meant these actors were sometimes side-lined (e.g., the MoHS in the transition to the NERC and DERC system; and local actors that were not compensated to perform day-to-day interventions or considered for employment in the first place). As explicitly raised by some respondents, this, in turn, meant that an opportunity to build capacity within public institutions and amongst local actors was missed.

Several respondents, for example, acknowledged that they felt the militaries’ intervention had probably saved lives, but in removing MoHS leadership over the response, left the institution un(der)empowered and un(der)prepared for future public health crises (i.e., in the state it was in prior to the outbreak) (NGO-C-N-10; NGO-C-N-7; GoSL-M-N-6; USG-C-N-3). For example, a GoSL civilian respondent (an MoHS employee, specifically) described how the outbreak was

*…like you found yourself in an ocean in a little canoe, and there are waves and a hurricane, and you are lucky to escape. Now [that] I know [it] is a hurricane, I would use a bigger boat, and I would [then know] everything that is involved [in surviving]. And I would go against the hurricane and be… self-reliant. And [we would have] a system you can be proud of, that the health system in this country can handle issues on [its] own (GoSL-C-N-26).*

The respondent’s statement acknowledges that the MoHS (“*a little canoe*”) was overwhelmed by the Ebola outbreak (“*a hurricane*”), but that through the experience of responding to the Ebola outbreak, there was the opportunity to learn (that they “*would [then know] everything that is involved [in surviving]*”) (GoSL-C-N-26). Had that occurred, the health system would be stronger, more resilient, and empowered (it would be able to “*handle issues on [its] own*”) (GoSL-C-N-26).

However, the respondent did not feel this had occurred:

*The military is a military and anything that is health related should be in health, you understand me? You cannot mix water and oil. [GoSL political leaders] should bring every [health] component within the [MoHS’] sector, and they should be able to respond to things. Because… now we have military interference in the public health emergency management… To be honest, what I believe is [that] the military is a separate institution on its own. You cannot… amalgamate it with other institutions like the [MoHS]… We don’t need the military any longer… I should have confidence and I should be restoring confidence in my medical teams (GoSL-C-N-26).*

Essentially, the respondent suggests that the militaries’ intervention in the Ebola response was an incursion on the MoHS’ professional territory, and therein, confidence was not built amongst the institution’s civilian employees. This and related issues with the militaries’ intervention were cited frequently by respondents, regardless of their research grouping: many (n = 57) thought the militaries performed activities that were either being done or could have been done by civilian actors, which a smaller number (n = 6) characterised as representing military mission-creep (one UN respondent recognised the difficulty of getting the “*right balance between the national authorities*” and military actors in the response, but also said of national actors that “*when they’re kind of lollygagging and not really helping… [then they should] get the fuck out of the way*” (UN-C-N-3)).

Importantly, “*mission creep*” (NGO-C-N-6) is something that was perceived to have occurred even after the outbreak had concluded. For example, in Sierra Leone, military roles were never fully divorced from post-outbreak response architecture, such as in the country’s post-epidemic infectious disease response team that is equally comprised of MoHS staff and RSLAF personnel (GoSL-M-N-15). In the UK, meanwhile, documents obtained under a Freedom of Information (FOI) request suggest some in the UK Ministry of Defence (MoD) consider the British Armed Forces’ intervention as not just unique but as a civil-military model for future humanitarian and disaster relief operations [33]. In the words of the report, the UK MoD argues that the British Armed Forces’ intervention in Sierra Leone—specifically their contributions to C2—was sufficiently unique and efficacious to the overall HMG mission that their deployment should “always be considered”.

While some respondents identified and criticised this phenomenon—for example, one HMG civilian respondent said training the British Armed Forces to work in this capacity would be “*a little bit like painting the Forth Bridge*” (HMG-C-I-11), inasmuch as the structure of the military means those trained would be quickly cycled into different positions—others acknowledged the dilemma that was represented by the MoHS’ failure to contain the Ebola epidemic and the underlying need for life-saving assistance regardless of how problematic the nature of it happened to be.

A GoSL civilian respondent (and senior response decision maker) effectively captured this when they asked:

*When you are in a crisis and people are dying, what are you going to say to that mother who lost her child?… I would like some of these arrogant and ignorant commentators to put themselves in the shoes of a mother whose child is dying, and four of her other children are already dead, her husband is dead, six of her extended family are dead… I would like these people who talked about the militarisation of the Ebola response to go and tell that woman ‘Your child is going to die because the only person who can help is a soldier, but we do not want to send a soldier’ (GoSL-C-N-17).*

This point indicates that others may find the militaries’ intervention to have been problematic, but that perhaps for many Sierra Leoneans, these concerns were insignificant when compared with potentially life-saving assistance to the overall response to the Ebola epidemic.

## Discussion

Respondents—in this article, primarily national and district level ones—typically characterised the militaries’ ‘systems software’ intervention within their civil-military centres as positive in both nature and effect. To respondents, this included the enabling environment the militaries were perceived to put in place; a clarity of purpose and mission focus that was engendered through their intervention; and an overall civil-military and military-military complementarity that was manifested.

These positive associations, though, are predicated on various factors. For example, many Sierra Leonean respondents spoke of their expectation that Britain—Sierra Leone’s colonial ruler—would intervene out of paternalistic obligation. The positive associations were also predicated on there having been an overwhelming public health crisis to respond to in the first place (i.e., simply, that the militaries’ intervention in Sierra Leone only occurred because a crisis arose, so without the crisis, there could be no praise). But the risk of Ebola emerging and escalating into an overwhelming outbreak resulted from a number of pre-existing factors. As argued by other scholars, these factors—including colonial rule as exacerbated by neoliberal reforms—include the un(der)empowerment of the health system and the weakening of other public institutions; systemic poverty; and historical distrust in public authority [28, 29, 34–38]. These factors (both hardware and software issues) make a country vulnerable to not only a heightened zoonotic risks, but also a poorer ability to contain an outbreak that does occur [ 37, 39–41]. Accordingly, following the emergence of Ebola in West Africa, Sierra Leone’s health system became quickly overwhelmed, national and international institutions failed to mount an adequate response, and local groups had little-to-no recognised capacities and were thus excluded from it [42, 43].

However, these institutional weaknesses were not found in all national sectors: following the 1991–2002 Sierra Leone Civil War, HMG—Sierra Leone’s prior colonial power and believing a strong and professional national army was the only way to prevent another slide into civil war—assisted Sierra Leone with the post-war reformation of the country’s armed services. The army was disbanded, rebuilt, financed, and professionalised [44]. In other words, it was not only capacitated in its systems hardware, but also its systems software (in fact, focus was primarily on the latter, as seen in concerted training and professionalisation efforts). Taken together, this represented “a comprehensive transformation” of the country’s armed forces, which was made sustainable through the HMG-funded and British Armed Forces-supported ISAT programme; indeed, the HMG-supported reform of Sierra Leone’s national army was considered so successful that it “is frequently seen as *the* example of SSR [i.e., security sector reform]” (emphasis in original) [44]. Meanwhile, over a similar period of time to the weakening of Sierra Leone’s health system and strengthening of its national army, the field of humanitarianism and public health was increasingly securitised [45–47]. Therefore, while the decision to deploy militaries to Sierra Leone’s Ebola epidemic may have appeared unusual, it was arguably normative (both for the foreseeable escalation of the crisis, for military actors to be considered an expedient and viable last resort, and for the UK specifically to have intervened as the country’s colonial ruler).

Further, as related to this political and economic history in Sierra Leone, the militaries’ intervention was not only normative in its origin, but was—to an extent—in its nature and effect as well: as seen in the data, public institutions and local actors were seen to fail (and at times, had their authority taken away); these institutions and actors did not then have their capacity built up during the response; and their authority over public health issues affecting Sierra Leone was not fully returned thereafter. The response’s ‘mission focus’ on ending the outbreak under a health security approach meant other health system needs (such as maternal health or efforts to tackle malaria) were disrupted and relegated [48]. Both the British Armed Forces and RSLAF, meanwhile, were seen by many respondents as strong and effective Ebola response partners. Further, they learned important lessons, and subsequently expanded their roles in subsequent public health emergency responses (for example as seen in response to the COVID-19 pandemic) [49]. This normalisation of military capabilities beyond warfighting is arguably concerning, especially in a country with a history of coups and recently out of civil war (this concern is noted in the wider literature on strengthening security structures in this way) [50–55].

In other words, while the lack of investment in public and civilian (infra)structures in Sierra Leone prior to the Ebola outbreak was itself an exacerbating factor that contributed to the origin of the outbreak (as is also highlighted by other scholars), the data presented in this article reveals how the militarised response—including for the various negative effects that respondents perceived in the way the militaries worked—were felt by some respondents to both maintain and contribute to perpetuation of these public sector weaknesses.

Nevertheless, despite these harms perceived by many respondents, a significant majority felt that the militaries’ intervention in Sierra Leone’s Ebola response represented life-saving assistance (not necessarily clinically so, but as in support of the wider Ebola response). Furthermore, if one argues—as most respondents did—that this assistance helped to shorten the overall duration of the outbreak (though this received wisdom is contested by some academics), [28, 29] the positive effects of the militaries’ intervention are further compounded. For example, shortening the outbreak (if this did indeed occur) would have conceivably helped to facilitate a return of childhood education, health services, and other public health campaigns that had been temporarily cancelled; removed various disruptions to personal and community livelihoods and thus re-opened economies; ended background stress, fear, stigma, and anxiety caused by the ongoing outbreak; and permitted billions of dollars of aid money committed to the Ebola response to be diverted to other public health causes (as was done for the subsequent Zika virus epidemic in Central and South America) [56].

Again, however, the fact the militaries’ intervention was required also underlines how such intervention can have deleterious long-term effects, if and when it is not structured in such a way that it builds capacities within public institutions and local actors, and/or is not followed by robust investment in health systems once the crisis has been contained (i.e., if the underlying status quo and vulnerabilities are allowed to remain intact). In short, a history of weakened public institutions and insufficient promotion of local populations in Sierra Leone was, to some extent, reiterated in and reinforced through the limited empowerment and inclusion of these actors within the militarised Ebola response.

**Fig. 3:**
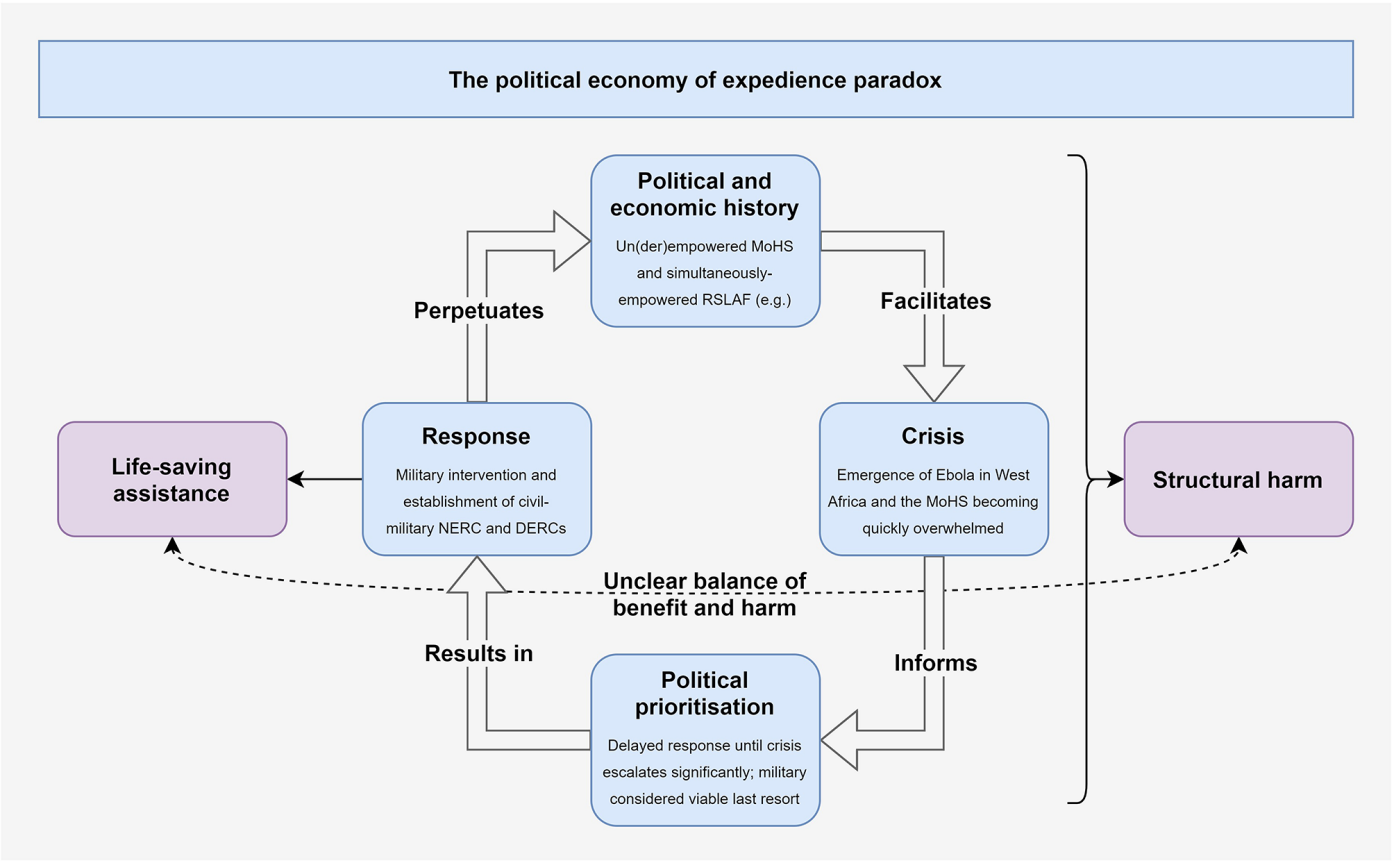
The political economy of expedience paradox as seen in the militarised response to Sierra Leone’s Ebola epidemic. (Source: author)

Taken together, this concept amounts to a paradox this article argues should be termed the ‘political economy of expedience’: the particular political and economic context that facilitates a given crisis; informs the ways it (and the response to it) are politically prioritised; results in the selection and manifestation of an expedient form of response (which delivers life-saving assistance while also exacerbating structural harms, the overall balance of which is unclear); and that finally, serves to further normalise or perpetuate the political economy that facilitated the crisis in the first place (Fig. 3).

## Conclusion

The UK and Sierra Leonean militaries’ intervention in Sierra Leone’s Ebola response removed the EOC’s civilian authority, and effected changes that were considered by a wide array of both civilian and military respondents to be very valuable.

However, the perceived value and efficacy of this militarised assistance was consequent to the apparent inability of the MoHS to effectively perform their core mandate—they were perceived to have insufficient ‘systems software’, and certainly had insufficient ‘systems hardware’ to deal with the crisis. Therein, the militaries’ deployment undermined public institutions and hindered capacity building, and once deployed, many Ebola-affected community members were unable to exercise their own more local forms of public authority in response to the crisis at hand. In other words, the militaries may have been considered admirable firefighters by many ERWs in Sierra Leone’s Ebola response, but their legacy is as ones who left embers behind. Those embers may not be material—indeed, this article focused primarily on the less tangible ways in which the militaries contributed (i.e., ‘systems software’), to include ways of working and organising, and mechanisms of coordination. Like the need to build capacity in the health ‘systems hardware’ by building new hospitals and ensuring robust supply chain, so too does a health system require capacity building in and an opportunity to exercise its ‘systems software’. In intervening militarily, that did not occur to nearly the same extent that might have otherwise occurred. That likely saved lives, but a gap filled ad-hoc remains a gap.

This article has argued that this paradox be termed the ‘political economy of expedience’—i.e., wherein the militaries were felt to provide life-saving assistance through their intervention in Sierra Leone’s Ebola response, but did so in a way that also caused (or at least insufficiently addressed) structural harm. This concept is an important contribution to the analysis of public health emergency response and resilience building that could inform the design of future responses so as to maximise the benefits and minimise the harms of exogenous interventions, therein mitigating the paradox.

## Data Availability

Data and materials are confidential.
